# 3,4-Dinitro-1*H*-pyrazole benzene 0.25-solvate

**DOI:** 10.1107/S1600536811015996

**Published:** 2011-05-11

**Authors:** Yong-Xiang Li, Shan Du, Jian-Long Wang

**Affiliations:** a School of Chemical Engineering and Environment, North University of China, Taiyuan, People’s Republic of China

## Abstract

The asymmetric unit of the title compound, 4C_3_H_2_N_2_O_4_·C_6_H_6_, contains two independent dinitro­pyrazole mol­ecules and half a benzene solvent mol­ecule, which lies on a crystallographic inversion centre. Each pyrazole ring is essentially planar (mean deviations of 0.009 and 0.002 Å), with the two nitro groups rotated out of the plane [dihedral angles = 11.7 (2)/31.1 (1) and 21.8 (2)/25.0 (1)° for the two mol­ecules].

## Related literature

For the biological properties of polynitro­pyrazoles, see: Alejandre-Durán *et al.* (1986 ▶); Grigor’ev *et al.* (1998) ▶; Xuan *et al.* (1999 ▶). For their detonation properties, see: Keshavarz *et al.* (2007 ▶); Zaitsev *et al.* (2009) ▶. For the synthesis, see: Katritzky *et al.* (2005 ▶). 
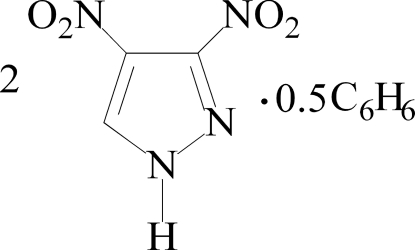


## Experimental

### 

#### Crystal data

2C_3_H_2_N_4_O_4_·0.5C_6_H_6_
*M* *_r_* = 355.23
Monoclinic, 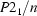

*a* = 7.4579 (15) Å
*b* = 9.787 (2) Å
*c* = 19.534 (4) Å
β = 94.87 (3)°
*V* = 1420.7 (5) Å^3^
*Z* = 4
Mo *K*α radiation
μ = 0.15 mm^−1^
*T* = 123 K
0.30 × 0.20 × 0.20 mm

#### Data collection

Rigaku R-AXIS RAPID IP diffractometer
Absorption correction: multi-scan (*ABSCOR*; Higashi, 1995 ▶) *T*_min_ = 0.957, *T*_max_ = 0.971
3454 measured reflections
3256 independent reflections
1267 reflections with *I* > 2σ(*I*)
*R*_int_ = 0.084

#### Refinement

*R*[*F* ^2^ > 2σ(*F* ^2^)] = 0.060
*wR*(*F* ^2^) = 0.105
*S* = 0.92
3256 reflections
235 parameters
2 restraints
H atoms treated by a mixture of independent and constrained refinement
Δρ_max_ = 0.27 e Å^−3^
Δρ_min_ = −0.33 e Å^−3^

### 

Data collection: *RAPID-AUTO* (Rigaku, 2000 ▶); cell refinement: *RAPID-AUTO*; data reduction: *CrystalStructure* (Rigaku, 2000 ▶); program(s) used to solve structure: *SHELXS97* (Sheldrick, 2008 ▶); program(s) used to refine structure: *SHELXL97* (Sheldrick, 2008 ▶); molecular graphics: *SHELXTL* (Sheldrick, 2008 ▶); software used to prepare material for publication: *SHELXL97*.

## Supplementary Material

Crystal structure: contains datablocks I, global. DOI: 10.1107/S1600536811015996/zs2110sup1.cif

Structure factors: contains datablocks I. DOI: 10.1107/S1600536811015996/zs2110Isup2.hkl

Supplementary material file. DOI: 10.1107/S1600536811015996/zs2110Isup3.cml

Additional supplementary materials:  crystallographic information; 3D view; checkCIF report

## Figures and Tables

**Fig. 1.**
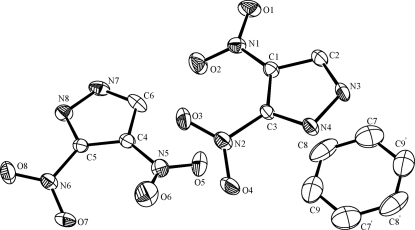
The molecular structure of the asymmetric unit of the title compound with the atom numbering scheme. The benzene molecule has inversion symmetry [symmetry code (i): -x + 2, -y, -z]. Hydrogen atoms are omitted and displacement ellipsoids are drawn at the 30% probability level.

## supplementary crystallographic information

### Comment

Polynitropyrazole systems have been investigated extensively because of their biological activity (Alejandre-Durán *et al.*, 1986; Grigor'ev *et al.*, 1998; Xuan *et al.*, 1999). Recently, these so called "high energy density materials" have attracted renewed attention because of their favorable detonation performance (Keshavarz *et al.*, 2007; Zaitsev *et al.*, 2009). As a potential candidate, 3,4-dinitropyrazole was synthesized by the nitration of pyrazole (Katritzky *et al.*, 2005). Here we report the crystal structure of the title compound, the benzene solvate 4(C_3_H_2_N_2_O_4_). C_6_H_6_ (I).

In the crystal structure of (I) (Fig. 1), the nitro groups are twisted with respect to the pyrazole plane, making dihedral angles of 11.7° (N1/O1, O2), 31.1° (N2/O3, O4) (molecule A) and 21.8° (N5/O5, O6), 25.0° (N6/O7, O8) (molecule B).

### Experimental

The title compound was prepared by the nitrification of pyrazole according to the literature method (Katritzky *et al.*, 2005). Single crystals suitable for X-ray diffraction were obtained by evaporation of a solution of the compound in benzene at room temperature.

### Refinement

All H atoms were positioned geometrically and treated as riding, with C—H bond lengths constrained to 0.95 Å and *U*_iso_(H) = 1.2*U*_eq_(C), and the N—H bond = 0.87 Å and *U*_iso_(H) = 1.5*U*_eq_(N).

### Crystal data

**Table:**

2C_3_H_2_N_4_O_4_·0.5C_6_H_6_	*F*(000) = 724
*M**_r_* = 355.23	*D*_x_ = 1.661 Mg m^−^^3^
Monoclinic, *P*2_1_/*n*	Mo *K*α radiation, λ = 0.71073 Å
Hall symbol: -P 2yn	Cell parameters from 3256 reflections
*a* = 7.4579 (15) Å	θ = 2.1–27.5°
*b* = 9.787 (2) Å	µ = 0.15 mm^−^^1^
*c* = 19.534 (4) Å	*T* = 123 K
β = 94.87 (3)°	Block, colorless
*V* = 1420.7 (5) Å^3^	0.30 × 0.20 × 0.20 mm
*Z* = 4	

### Data collection

**Table:**

Rigaku R-AXIS RAPID IP diffractometer	3256 independent reflections
Radiation source: fine-focus sealed tube	1267 reflections with *I* > 2σ(*I*)
graphite	*R*_int_ = 0.084
Detector resolution: 10.00 pixels mm^-1^	θ_max_ = 27.5°, θ_min_ = 2.1°
ω scans	*h* = −9→9
Absorption correction: multi-scan (*ABSCOR*; Higashi, 1995)	*k* = −12→12
*T*_min_ = 0.957, *T*_max_ = 0.971	*l* = −25→25
3256 measured reflections	

### Refinement

**Table:**

Refinement on *F*^2^	Secondary atom site location: difference Fourier map
Least-squares matrix: full	Hydrogen site location: inferred from neighbouring sites
*R*[*F*^2^ > 2σ(*F*^2^)] = 0.060	H atoms treated by a mixture of independent and constrained refinement
*wR*(*F*^2^) = 0.105	*w* = 1/[σ^2^(*F*_o_^2^) + (0.*P*)^2^] where *P* = (*F*_o_^2^ + 2*F*_c_^2^)/3
*S* = 0.92	(Δ/σ)_max_ < 0.001
3256 reflections	Δρ_max_ = 0.27 e Å^−^^3^
235 parameters	Δρ_min_ = −0.33 e Å^−^^3^
2 restraints	Extinction correction: *SHELXL97* (Sheldrick, 2008), Fc^*^=kFc[1+0.001xFc^2^λ^3^/sin(2θ)]^-1/4^
Primary atom site location: structure-invariant direct methods	Extinction coefficient: 0.0081 (6)

### Special details

**Table:**

Geometry. All e.s.d.'s (except the e.s.d. in the dihedral angle between two l.s. planes) are estimated using the full covariance matrix. The cell e.s.d.'s are taken into account individually in the estimation of e.s.d.'s in distances, angles and torsion angles; correlations between e.s.d.'s in cell parameters are only used when they are defined by crystal symmetry. An approximate (isotropic) treatment of cell e.s.d.'s is used for estimating e.s.d.'s involving l.s. planes.
Refinement. Refinement of *F*^2^ against ALL reflections. The weighted *R*-factor *wR* and goodness of fit *S* are based on *F*^2^, conventional *R*-factors *R* are based on *F*, with *F* set to zero for negative *F*^2^. The threshold expression of *F*^2^ > σ(*F*^2^) is used only for calculating *R*-factors(gt) *etc*. and is not relevant to the choice of reflections for refinement. *R*-factors based on *F*^2^ are statistically about twice as large as those based on *F*, and *R*- factors based on ALL data will be even larger.

### Fractional atomic coordinates and isotropic or equivalent isotropic displacement parameters (Å^2^)

**Table:**

	*x*	*y*	*z*	*U*_iso_*/*U*_eq_	
C1	0.9887 (4)	0.1907 (3)	0.19586 (16)	0.0327 (8)	
C2	1.1357 (4)	0.1088 (4)	0.19047 (17)	0.0420 (9)	
H2	1.2585	0.1359	0.1953	0.050*	
C3	0.8410 (4)	0.1014 (3)	0.18468 (16)	0.0289 (8)	
C4	0.5086 (4)	0.5510 (3)	0.09984 (17)	0.0387 (9)	
C5	0.3685 (4)	0.6383 (3)	0.11466 (16)	0.0344 (8)	
C6	0.6606 (4)	0.6268 (4)	0.10959 (18)	0.0538 (11)	
H6	0.7801	0.5975	0.1045	0.065*	
C7	1.1648 (8)	0.0615 (7)	0.0108 (2)	0.0902 (16)	
H7	1.2790	0.1045	0.0180	0.108*	
C8	1.0067 (11)	0.1372 (5)	0.0150 (2)	0.0921 (19)	
H8	1.0132	0.2318	0.0258	0.111*	
C9	0.8469 (8)	0.0760 (7)	0.0040 (2)	0.0880 (17)	
H9	0.7399	0.1279	0.0063	0.106*	
N1	0.9970 (4)	0.3361 (3)	0.20565 (14)	0.0409 (7)	
N2	0.6477 (3)	0.1259 (3)	0.18791 (15)	0.0431 (8)	
N3	1.0721 (3)	−0.0172 (3)	0.17704 (15)	0.0415 (8)	
N4	0.8896 (3)	−0.0246 (3)	0.17325 (13)	0.0364 (7)	
N5	0.5049 (4)	0.4072 (3)	0.08235 (17)	0.0540 (9)	
N6	0.1748 (3)	0.6197 (3)	0.10954 (15)	0.0422 (7)	
N7	0.6079 (4)	0.7520 (4)	0.12796 (17)	0.0533 (9)	
N8	0.4295 (3)	0.7621 (3)	0.13217 (14)	0.0455 (8)	
O1	1.1467 (3)	0.3853 (3)	0.22096 (13)	0.0593 (8)	
O2	0.8580 (3)	0.4027 (3)	0.19682 (14)	0.0638 (8)	
O3	0.6020 (3)	0.2140 (3)	0.22658 (13)	0.0616 (8)	
O4	0.5471 (3)	0.0518 (3)	0.15166 (14)	0.0585 (8)	
O5	0.6340 (4)	0.3642 (3)	0.05424 (15)	0.0840 (10)	
O6	0.3762 (4)	0.3390 (3)	0.09674 (17)	0.0864 (10)	
O7	0.1130 (3)	0.5307 (3)	0.07052 (13)	0.0636 (8)	
O8	0.0861 (3)	0.6953 (3)	0.14284 (14)	0.0631 (8)	
H7A	0.684 (5)	0.820 (4)	0.127 (3)	0.17 (2)*	
H3	1.138 (3)	−0.091 (2)	0.1735 (17)	0.052 (11)*	

### Atomic displacement parameters (Å^2^)

**Table:**

	*U*^11^	*U*^22^	*U*^33^	*U*^12^	*U*^13^	*U*^23^
C1	0.0275 (18)	0.0321 (19)	0.038 (2)	−0.0016 (15)	0.0019 (15)	0.0026 (17)
C2	0.0259 (18)	0.042 (2)	0.057 (2)	0.0014 (17)	−0.0032 (16)	−0.004 (2)
C3	0.0226 (16)	0.0290 (18)	0.0343 (18)	0.0030 (14)	−0.0015 (14)	0.0029 (16)
C4	0.0326 (19)	0.039 (2)	0.044 (2)	0.0008 (17)	−0.0042 (15)	−0.001 (2)
C5	0.0306 (18)	0.034 (2)	0.038 (2)	−0.0019 (16)	−0.0017 (14)	−0.0027 (18)
C6	0.0279 (19)	0.070 (3)	0.063 (3)	−0.001 (2)	−0.0027 (18)	0.013 (3)
C7	0.112 (5)	0.101 (5)	0.056 (3)	−0.015 (4)	−0.007 (3)	0.007 (3)
C8	0.177 (6)	0.047 (3)	0.047 (3)	−0.006 (4)	−0.019 (4)	−0.001 (3)
C9	0.127 (5)	0.089 (5)	0.046 (3)	0.043 (4)	−0.004 (3)	−0.002 (3)
N1	0.0403 (18)	0.0392 (19)	0.0422 (18)	−0.0050 (15)	−0.0027 (14)	−0.0002 (16)
N2	0.0299 (16)	0.0406 (19)	0.058 (2)	−0.0009 (15)	−0.0004 (15)	0.0044 (18)
N3	0.0257 (15)	0.042 (2)	0.056 (2)	0.0115 (15)	−0.0010 (13)	−0.0006 (17)
N4	0.0232 (14)	0.0343 (17)	0.0511 (18)	0.0016 (12)	0.0007 (12)	0.0042 (15)
N5	0.051 (2)	0.048 (2)	0.061 (2)	0.0108 (18)	−0.0078 (17)	−0.0066 (19)
N6	0.0311 (16)	0.0382 (18)	0.056 (2)	−0.0012 (15)	−0.0003 (14)	0.0049 (18)
N7	0.045 (2)	0.049 (2)	0.063 (2)	−0.0178 (18)	−0.0099 (16)	0.0041 (19)
N8	0.0431 (18)	0.0332 (18)	0.059 (2)	−0.0049 (15)	−0.0009 (15)	−0.0002 (17)
O1	0.0446 (15)	0.0533 (17)	0.0775 (19)	−0.0211 (14)	−0.0100 (13)	−0.0038 (16)
O2	0.0497 (15)	0.0403 (16)	0.099 (2)	0.0119 (13)	−0.0072 (15)	0.0013 (16)
O3	0.0338 (14)	0.0637 (19)	0.089 (2)	0.0096 (14)	0.0136 (13)	−0.0253 (18)
O4	0.0272 (13)	0.0502 (17)	0.096 (2)	−0.0024 (12)	−0.0094 (12)	−0.0134 (17)
O5	0.071 (2)	0.078 (2)	0.105 (2)	0.0246 (17)	0.0199 (17)	−0.030 (2)
O6	0.082 (2)	0.0369 (18)	0.141 (3)	−0.0096 (16)	0.015 (2)	−0.0024 (19)
O7	0.0371 (14)	0.066 (2)	0.085 (2)	−0.0130 (14)	−0.0100 (13)	−0.0203 (18)
O8	0.0405 (15)	0.0539 (18)	0.098 (2)	0.0059 (14)	0.0231 (14)	−0.0095 (18)

### Geometric parameters (Å, °)

**Table:**

C1—C2	1.370 (4)	C8—C9	1.335 (7)
C1—C3	1.409 (4)	C8—H8	0.9500
C1—N1	1.436 (4)	C9—C7^i^	1.377 (7)
C2—N3	1.339 (4)	C9—H9	0.9500
C2—H2	0.9500	N1—O2	1.224 (3)
C3—N4	1.310 (4)	N1—O1	1.229 (3)
C3—N2	1.468 (3)	N2—O3	1.214 (3)
C4—C6	1.355 (4)	N2—O4	1.225 (3)
C4—C5	1.398 (4)	N3—N4	1.359 (3)
C4—N5	1.448 (4)	N3—H3	0.876 (10)
C5—N8	1.328 (4)	N5—O6	1.221 (4)
C5—N6	1.451 (4)	N5—O5	1.223 (4)
C6—N7	1.344 (4)	N6—O8	1.217 (3)
C6—H6	0.9500	N6—O7	1.222 (3)
C7—C9^i^	1.377 (7)	N7—N8	1.344 (4)
C7—C8	1.401 (7)	N7—H7A	0.878 (10)
C7—H7	0.9500		
			
C2—C1—C3	104.2 (3)	C8—C9—C7^i^	120.8 (5)
C2—C1—N1	124.3 (3)	C8—C9—H9	119.6
C3—C1—N1	131.3 (3)	C7^i^—C9—H9	119.6
N3—C2—C1	106.3 (3)	O2—N1—O1	124.5 (3)
N3—C2—H2	126.8	O2—N1—C1	118.8 (3)
C1—C2—H2	126.8	O1—N1—C1	116.6 (3)
N4—C3—C1	112.7 (3)	O3—N2—O4	126.1 (3)
N4—C3—N2	116.7 (3)	O3—N2—C3	118.1 (3)
C1—C3—N2	130.5 (3)	O4—N2—C3	115.7 (3)
C6—C4—C5	105.5 (3)	C2—N3—N4	113.3 (3)
C6—C4—N5	124.4 (3)	C2—N3—H3	125 (2)
C5—C4—N5	130.0 (3)	N4—N3—H3	121 (2)
N8—C5—C4	111.4 (3)	C3—N4—N3	103.4 (3)
N8—C5—N6	116.7 (3)	O6—N5—O5	125.4 (3)
C4—C5—N6	131.7 (3)	O6—N5—C4	118.5 (3)
N7—C6—C4	106.1 (3)	O5—N5—C4	116.1 (3)
N7—C6—H6	127.0	O8—N6—O7	125.0 (3)
C4—C6—H6	127.0	O8—N6—C5	118.0 (3)
C9^i^—C7—C8	119.4 (5)	O7—N6—C5	117.0 (3)
C9^i^—C7—H7	120.3	N8—N7—C6	113.4 (3)
C8—C7—H7	120.3	N8—N7—H7A	126 (3)
C9—C8—C7	119.8 (5)	C6—N7—H7A	119 (3)
C9—C8—H8	120.1	C5—N8—N7	103.7 (3)
C7—C8—H8	120.1		
			
C3—C1—C2—N3	0.1 (4)	C1—C3—N2—O3	−29.0 (5)
N1—C1—C2—N3	−175.8 (3)	N4—C3—N2—O4	−31.6 (4)
C2—C1—C3—N4	−0.1 (4)	C1—C3—N2—O4	152.5 (3)
N1—C1—C3—N4	175.4 (3)	C1—C2—N3—N4	−0.1 (4)
C2—C1—C3—N2	175.9 (3)	C1—C3—N4—N3	0.0 (4)
N1—C1—C3—N2	−8.6 (6)	N2—C3—N4—N3	−176.6 (3)
C6—C4—C5—N8	0.5 (4)	C2—N3—N4—C3	0.1 (4)
N5—C4—C5—N8	176.4 (4)	C6—C4—N5—O6	155.9 (4)
C6—C4—C5—N6	175.6 (3)	C5—C4—N5—O6	−19.3 (6)
N5—C4—C5—N6	−8.5 (6)	C6—C4—N5—O5	−23.7 (5)
C5—C4—C6—N7	−1.0 (4)	C5—C4—N5—O5	161.0 (3)
N5—C4—C6—N7	−177.2 (3)	N8—C5—N6—O8	−25.2 (4)
C9^i^—C7—C8—C9	−0.8 (8)	C4—C5—N6—O8	159.9 (3)
C7—C8—C9—C7^i^	0.8 (9)	N8—C5—N6—O7	153.1 (3)
C2—C1—N1—O2	166.3 (3)	C4—C5—N6—O7	−21.8 (5)
C3—C1—N1—O2	−8.5 (6)	C4—C6—N7—N8	1.2 (4)
C2—C1—N1—O1	−12.0 (5)	C4—C5—N8—N7	0.3 (4)
C3—C1—N1—O1	173.2 (3)	N6—C5—N8—N7	−175.7 (3)
N4—C3—N2—O3	146.8 (3)	C6—N7—N8—C5	−0.9 (4)

Symmetry codes: (i) −*x*+2, −*y*, −*z*.

## References

[bb1] Alejandre-Durán, E., Ruiz-Rubio, M., Claramunt, R. M., López, C. & Pueyo, C. (1986). *Environ. Mutagen.* **8**, 611–619. 10.1002/em.2860080411 3525137

[bb2] Grigor’ev, N. B., Kalinkina, M. A., Chechekin, G. V., Nikitin, V. B. & Engalycheva, G. N. (1998). *Pharm. Chem. J.* **32**, 127–131.

[bb3] Higashi, T. (1995). ABSCOR. Rigaku Corporation, Tokyo, Japan.

[bb4] Katritzky, A. R., Scriven, E. F. V., Majumder, S., Akhmedova, R. G., Akhmedov, N. G. & Vakulenko, A. V. (2005). *ARKIVOC*, **iii**, 179–191.

[bb5] Keshavarz, M. H., Pouretedal, H. R. & Semnani, A. (2007). *J. Hazard. Mater* **141**, 803–807. 10.1016/j.jhazmat.2006.07.046 16956725

[bb6] Rigaku (2000). *RAPID-AUTO* and *CrystalStructure* Rigaku Corporation, Tokyo, Japan.

[bb7] Sheldrick, G. M. (2008). *Acta Cryst.* A**64**, 112–122. 10.1107/S0108767307043930 18156677

[bb8] Xuan, B., Zhou, Y. H. & Verma, R. S. (1999). *J. Ocul. Pharmacol. Ther* **15**, 135–142. 10.1089/jop.1999.15.135 10229491

[bb9] Zaitsev, A. A., Dalinger, I. L. & Shaevelev, S. A. (2009). *Russ. Chem. Rev.* **78**, 589–627.

